# Comparison of DP3 Signals Evoked by Comfortable 3D Images and 2D Images — an Event-Related Potential Study using an Oddball Task

**DOI:** 10.1038/srep43110

**Published:** 2017-02-22

**Authors:** Peng Ye, Xiang Wu, Dingguo Gao, Haowen Liang, Jiahui Wang, Shaozhi Deng, Ningsheng Xu, Juncong She, Jun Chen

**Affiliations:** 1 State Key Laboratory of Optoelectronic Materials and Technologies, Guangdong Province Key Laboratory of Display Material and Technology, School of Electronics and Information Technology, Sun Yat-sen University, Guangzhou, China; 2 State Key Laboratory of Optoelectronic Materials and Technologies, Sun Yat-Sen University, Guangzhou, China; 3 Department of Psychology, Sun Yat-Sen University, Guangzhou, China

## Abstract

The horizontal binocular disparity is a critical factor for the visual fatigue induced by watching stereoscopic TVs. Stereoscopic images that possess the disparity within the ‘comfort zones’ and remain still in the depth direction are considered comfortable to the viewers as 2D images. However, the difference in brain activities between processing such comfortable stereoscopic images and 2D images is still less studied. The DP3 (differential P3) signal refers to an event-related potential (ERP) component indicating attentional processes, which is typically evoked by odd target stimuli among standard stimuli in an oddball task. The present study found that the DP3 signal elicited by the comfortable 3D images exhibits the delayed peak latency and enhanced peak amplitude over the anterior and central scalp regions compared to the 2D images. The finding suggests that compared to the processing of the 2D images, more attentional resources are involved in the processing of the stereoscopic images even though they are subjectively comfortable.

Stereoscopic-3D (hereinafter referred to as 3D for simplicity) display technology has drawn a lot of attention because of its greater sense of immersion and presence compared to the conventional 2D display. It works as presenting paired images with a horizontal binocular disparity into each eye and the visual system fuses the images to get the depth information. Unfortunately, viewers are reported prone to fatigue after watching 3D displays^1^, as indicated by subjectively reported general tiredness^2^,^3^ or the decay of ocular performance^4^. There are several potential causes of this induced visual fatigue^5^,^6^,^7^,^8^,^9^,^10^,^11^, among which the accommodation-vergence conflict is principally present in all kinds of flat panel 3D displays. When watching 3D contents with a disparity on 3D displays, the eye accommodation would tend to prefer the position of the screen to obtain a clear image while the vergence would tend to prefer the position of the virtually reconstructed object out of the screen to maintain a single fusion image. This could cause a conflict of the coupled ocular system that does not occur in natural viewing conditions^12^,^13^,^14^. The excessive binocular disparity would enlarge this conflict and is thus thought to be a critical factor of the visual fatigue induced by watching the 3D displays^8^,^15^. Fortunately, the human visual system is tolerant of a small amount of blurring of images on the retina and a single fused percept could be obtained for objects located within a small region in front of and behind the fixation plane, because of the ocular characteristics of the depth of focus and the Panum’s fusional area. The accommodation-vergence conflict could therefore be easily tolerated by the visual system if the disparity of the 3D contents was not too large^16^. The ‘comfort zone’ has been proposed to limit the horizontal binocular disparity within a certain range, in which the visual fatigue due to the conflict is modest and tolerable^5^,^16^. Several existing comfort zones and a comparison of them could be found in a review by W. J. Tam^16^. Besides the excessive disparity, the change of the disparity that could be perceived as the movement of the virtual objects in the depth direction was also reported as an important factor inducing the visual fatigue^6^,^7^,^15^, even within the ‘comfort zone’^2^,^8^,^17^. Therefore, it could be inferred that the stereoscopic images that possess a small disparity within the ‘comfort zone’ and remain still in the depth direction would be perceived relatively comfortable, which could be a useful guidance in the production of 3D contents. In supporting of this, such 3D images have been found to be as comfortable to viewers as the 2D images when tested before and after a period of viewing based on subjective or ocular measurements^2^.

Although the 3D images that possess a small disparity within the ‘comfort zone’ and remain still in the depth direction are considered to be as comfortable to viewers as the 2D images, the brain processing may differ between viewing the comfortable 3D images and 2D images. Also importantly for the production of 3D contents, the real-time brain processing when viewers watching 3D contents should be assessed, in addition to the measurements before and after a period of viewing. The electroencephalography (EEG) is one of the most often used brain-imaging approaches in brain-computer interface research. Some studies show that after watching a period of 3D movies the accumulative visual fatigue could cause changes of EEG-related signals, such as the energy in certain wavebands^18^, the steady-state evoked potential (SSVEP) or the P600 (a delayed member of the P3 component)^19^. For real-time measurements, some studies have found that small horizontal disparities did have influences on early event-related potential (ERP) components^20^,^21^,^22^,^23^,^24^, such as P1^20^, N1^23^,^24^, P2^24^ and N2^23^. The later ERP components, however, have been less investigated when watching the comfortable 3D images.

The DP3 (differential P3) signal is one of the most studied later ERP components, and is typically obtained in an oddball task by subtracting the P3 in response to the standard stimuli from the P3 in response to the odd target stimuli^25^,^26^. The oddball task is designed to investigate the processes associated with attending to the odd target and the DP3 signal is particularly suitable for the examination of the underlying high-level attentional functions, relative to the low-level sensory functions occurring at an early processing stage. For the example of the current 3D vs. 2D topic, because the 3D and 2D conditions are different viewing conditions, the 3D and 2D stimuli would differ in low-level physical features (e.g., in the present study, the digits were depicted by lines in the 2D condition and were only seen via binocularly fusion of monocular information in the 3D condition. See the Methods below). Such physical differences between the 3D and 2D stimuli would result in differences between the ERPs in response to the 3D and 2D stimuli. However, the physical features are the same for the deviant and standard stimuli, in either the 3D or 2D condition; and the effects of the physical features are supposed to be controlled in the differential waveforms. As Luck has stated, such sensory confounds can be eliminated by computing “rare-minus-frequent difference waves separately” for the 3D and 2D stimuli, and the “differences between these difference waves cannot be attributed to pure sensory differences” between the 3D and 2D stimuli^27^. Therefore, the difference between the DP3 signals in the 3D and 2D conditions cannot be attributed to low-level brain processing that is related to pure sensory differences between the 3D and 2D stimuli; instead, it reflects high-level brain processing underlying the interaction between the 3D vs. 2D viewing and attending to the target (which is the investigation aim of the present study). In general, the DP3 is suggested to reflect high-level attentional processing^25^,^26^. Specifically, the latency of the DP3 is suggested to be associated with the evaluation of the target stimuli, and would increase as categorization of target stimuli becomes more difficult^28^,^29^. The amplitude of the DP3 is found to be proportional to the amount of attentional resources allocated to a given target stimulus^30^ and is suggested to reflect the demands of cognitive resources of the task^29^. It remains unclear whether the high-level attentional processing as indicated by the DP3 signal differs between watching comfortable 3D images and the 2D images. As introduced above, the fatigue of watching 3D displays is usually indicated by subjectively reported tiredness^2^,^3^. For the underlying mechanisms, while previous studies have majorly examined the early low-level processing^20^,^21^,^22^,^23^,^24^, the role of the high-level attentional processing in the 3D-related fatigue has been less investigated.

Therefore, the present study was designed to investigate the difference between the DP3 signals when watching the comfortable 3D images that possessed a disparity within the ‘comfort zones’ and remained still in the depth direction and the 2D images. The subjects watched 3D or 2D images while performing an oddball task in which the subjects were required to respond to the presented digits by pressing corresponding keys (Fig. 1). In the 2D condition the digits and the interval fixation cross were perceived as flat on the screen; and in the 3D condition the images possessed a crossed disparity of 0.6 arc deg that was well within the ‘comfort zones’^16^, and the digits and the interval fixation cross were perceived as standing in front of the screen. The change of the disparity in the stimulus sequence of the 3D condition was carefully controlled in the present study by the design of the same disparity for the digits and the interval fixation cross. All of the images in the stimulus sequence possessed the same disparity, thus assuring that the 3D digits in a sequence were perceived as staying still in depth. The EEG was recorded when the subjects performed the tasks and the subjective scores concerning the general feeling of comfort were obtained before and after the tasks.

The analyses were focused on the latency and amplitude of the DP3 signal. In addition, preceding to the DP3 signal, some studies have observed an earlier DN2 signal (obtained by subtracting the N2 in response to the standard stimuli from the N2 in response to the odd target stimuli), which may be also related to attentional resource allocation^31^ and was thus also examined in the present study. We proposed that watching the comfortable 3D images may involve more attentional processing, compared to watching the 2D images. Specifically, the delayed latency and enhanced amplitude of the DP3 signal for watching the 3D than for the 2D images were predicted, which would reflect the difference in the attention-related brain processing between the two display conditions.

## Results

All of the subjects responded with high accuracy in both conditions [for the 3D condition: 91.16% for deviant stimuli and 96.97% for standard stimuli; for the 2D condition: 95.43% for deviant stimuli and 99.27% for standard stimuli], indicating that they were well engaged in the tasks. The subjective scores before and after the tasks were collected and their difference, i.e., the differential score, was calculated as the indicator of the induced visual fatigue^19^. The paired t-test showed that the differential score was not significantly different between the 2D (mean difference = 0.95, SD = 0.83) and 3D (mean difference = 0.9, SD = 0.91) conditions, though large standard deviations were observed in both conditions. In addition, the null result of the t-test was verified by calculating the Bayes Factor using a scaling factor of 0.707^32^, which revealed the strong evidence for the null hypothesis with a Scaled JZS Bayes Factor of 3.14. The result confirmed the success of the current design of the comfortable 3D images. A potential order effect was examined by separating the 20 subjects into two groups of ten subjects, one conducting the 3D task first and one conducting the 2D task first. There was no statistical difference in the differential subjective scores between the two groups. A two-way repeated measures analysis of variance (ANOVA) with two factors of dimension (3D, 2D) and stimulus type (deviant, standard) was conducted for the reaction times (RT). The main effect of stimulus type [F(1, 19) = 74.4, P < 0.005, η^2^ = 0.797] was significant, showing the typical oddball effect (567.1 ms and 505.9 ms for the deviant and standard stimuli, respectively). The main effect of dimension [F(1, 19) = 14.1, P = 0.001, η^2^ = 0.425] was significant, indicating that longer time was needed in responding to the stimuli in the 3D condition (563.8 ms and 509.2 ms for the 3D and 2D conditions, respectively). The interaction between the two factors was not significant.

**Figure 2:**
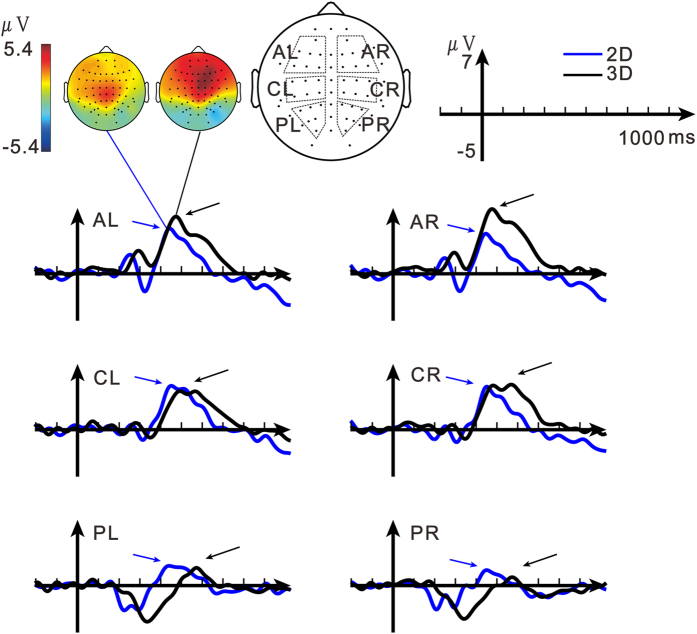
Illustration of the average differential waveforms (deviant vs. standard stimuli) in the 2D (blue) and 3D (black) conditions. The DP3 signal is indicated by the arrow. The topographies of the DP3 signals are plotted above the waveforms. Data are clustered into 3 scalp regions (anterior (A), central (C), posterior (P)) in each hemisphere, as shown on the right of the topographies.

For ERP results, the present study focused on the differential waveforms (deviant vs. standard stimuli) that revealed the DP3 signal. The waveforms for the deviant and standard stimuli are presented in Supplementary Fig. S1. The DP3 signals in the 2D and 3D conditions are shown in Fig. 2. For the purpose of statistical analyses, electrodes over the scalp were clustered into three regions in each hemisphere^33^. In line with previous results, the DP3 signal peaked at approximately 450 ms with a center scalp distribution^26^,^34^. Visual inspection indicated that, compared to the 2D condition, the DP3 signal in the 3D condition was of longer peak latency and had larger peak amplitude over the anterior and central scalp regions. The ANOVA with 3 factors of dimension (2D, 3D), region (anterior (A), central (C), posterior (P)) and hemisphere (left, right) was conducted for the latency and amplitude of the DP3 signals, respectively.

### Latency of the DP3 signal

The main effect of dimension [F(1, 7) = 12.02, P = 0.01,η^2^ = 0.632] was significant, indicating that the evaluation of the deviant vs. standard stimuli was more difficult in the 3D than in the 2D condition. The mean latency values and corresponding post-hoc comparisons between the 2D and 3D conditions are listed in Table 1, revealing that the delayed DP3 latency for the 3D than for the 2D condition was mainly over anterior and central scalp regions. Note that this strong effect was not only found on the group level, but was also evident in all individual subjects’ results (Supplementary Table S1). Other main effects and interactions were not significant.

### Amplitude of the DP3 signal

The main effect of region [F(2, 14) = 12.23, P = 0.003, η^2^ = 0.636], the interaction of region × hemisphere [F(2, 14) = 4.33, P = 0.038, η^2^ = 0.382] and the interaction of region × hemisphere × dimension [F(2, 14) = 5.71, P = 0.027, η^2^ = 0.449] were significant. The interaction of region × dimension [F(2, 14) = 4.77, P = 0.053, η^2^ = 0.405] was marginally significant. No other effect was found significant. The dimension related effects were the focus of the analyses. The mean amplitude values and the corresponding post-hoc comparisons between the 2D and 3D conditions are listed in Table 1, showing that the DP3 amplitude was enhanced in the 3D condition in the anterior and central regions, seemingly with a right lateralization.

### Latency and amplitude of the trough before the DP3 signal

There was a trough right before the DP3 signal, may representing the DN2 signal^31^ (Fig. 2). Visual inspection indicated that the results of the trough were generally similar to the results of the DP3, i.e., the trough in the 3D condition was also of longer peak latency and had larger peak amplitude over anterior scalp regions, compared to the 2D condition. These were confirmed in the analyses of the latency and amplitude of the trough. In the ANOVA results, the latency of the trough showed significant main effect of dimension [F(2, 14) = 16.41, P = 0.005, η^2^ = 0.701]. For the amplitude, the main effect of region [F(2, 14) = 10.55, P = 0.006, η^2^ = 0.601] and the interaction of region × dimension [F(2, 14) = 11.47, P = 0.003, η^2^ = 0.621] were significant. The mean values of the latency and amplitude of the 2D and 3D conditions for each region and the corresponding post-hoc comparisons between the 2D and 3D conditions are listed in Table 2. Compared to the enhanced DP3 amplitude in the 3D condition over mainly right anterior and central scalp regions, the enhanced amplitude of the trough was observed over mainly bilateral anterior regions.

## Discussion

The present study has compared the DP3 signals elicited by the 3D contents that possessed a disparity within the ‘comfort zones’ and remained still in the depth direction with the DP3 signals elicited by the 2D contents. The subjective measurement confirmed that the 3D contents were of similar comfort to the subjects as the 2D contents. The DP3 signal in the 3D condition showed the delayed latency and enhanced amplitude over anterior and central scalp regions compared to the 2D condition.

The excessive disparity and the disparity change in the depth direction of the 3D contents are two important factors inducing visual fatigue to the viewers^1^,^2^,^5^,^8^. In the present study, a moderate disparity of 0.6 arc deg was employed which is well within the ‘comfort zones’^16^. In addition, the image sequence of the 3D condition was designed to be perceived as remaining still in the depth direction by assigning the same disparity to the digit stimuli and the interval fixation cross. The subjective differential scores that reflected the induced visual fatigue was not significantly different between the 2D and 3D conditions, indicating the validity of the current design of the comfortable 3D contents.

Though the 3D contents were subjectively reported to be as comfortable as the 2D contents, the DP3 signals in the 2D and 3D conditions were revealed to be different. Compared to the 2D condition, the latency of the DP3 signal in the 3D condition was longer over mainly the anterior and central scalp electrodes, suggesting that it could be more difficult for evaluating the stimuli in the 3D condition^28^. The amplitude of the DP3 signal in the 3D condition was enhanced over mainly the right anterior and central scalp electrodes, suggesting that the task in the 3D condition may demand more attentional resources^35^,^36^,^37^. This is consistent with the observation that the DP3 amplitude decreases at the anterior and central electrode sites when fewer resources are involved^35^,^36^,^37^. The delayed latency and enhanced amplitude of the DP3 signal in the 3D condition compared to the 2D condition supported a view that the attentional processing indicated as the late ERP component DP3 is different for viewing the 3D and 2D contents, even though the 3D contents could be as comfortable as the 2D contents to the viewers.

In addition, there was a trough occurring right before the DP3 signal in the differential waveforms, which may be associated with the DN2 signal that has also been considered to reflect attentional resource allocation^31^. The results of the trough were basically similar to that of the DP3 signal, exhibiting longer latency and enhanced amplitude over the anterior scalp regions in the 3D compared to the 2D condition. This seemed to suggest that the attentional processing involved in watching the comfortable 3D contents as indicated by the DP3 signal may occur earlier, during the stage indicated by the trough. Therefore, the data of both the DP3 signal and the preceding trough indicated that more attentional resources were involved in the 3D than in the 2D condition, though it needs to be further examined in further research whether the results of the trough could be as reliable as the results of the DP3^38^. Moreover, the digit stimuli were used in this study and the current finding would be further verified in future studies by employing images of objects that have more 3D relevance.

## Conclusion

In the field of 3D display, a key issue is to determine whether the 3D contents within the comfort zone are comfortable to the viewers, particularly regarding objective measures (relative to subjective measures) of high-level cognitive brain functions (relative to low-level functions at sensory cortices). To address this issue, two important designs were made in the present study (see the Introduction for detailed discussion relative to previous studies). (1) An oddball task and the ERP DP3 signal were adopted to evaluate the high level attentional processing when viewing the 3D and 2D contents. (2) The change of the binocular disparity was carefully controlled in the 3D condition so that the 3D contents would be more comfortable to the viewers. With such designs, the present results showed that even though viewing the 3D and 2D contents were of similar subjective comfort, the former involved more demands of attentional resources, as indicated by the delayed latency and enhanced amplitude of the DP3 signal over the anterior and central scalp regions. Therefore, the current study makes both theoretical and methodological contributions to the field. Not only the results reveals a novel finding that the 3D contents within the comfort zone may not be comfortable to the viewers regarding attentional processing, but also the current work provides an effective ERP approach to sensitively assess the differences in attention-related brain functions between viewing the comfortable 3D and 2D contents.

## Materials and Methods

### Subjects

20 university students (13 males, age (mean = 24.1, SD = 2.2)) participated in the subjective measurement, and the ERPs were recorded from 8 of the 20 subjects [4 males, age (mean = 23.6, SD=2.1)]. All subjects were right-handed, and had normal or corrected to normal vision. All subjects had normal stereoscopic sensitivity. All subjects were required to take a good rest before the experiment. The written informed consent was obtained for each subject after a full explanation of the experiment. The research protocols of this study were approved by Psychology Department and State Key Laboratory of Optoelectronic Materials and Technologies, Sun Yat-Sen University. The methods were carried out in accordance with the approved guidelines.

### Stimuli

**Figure 3:**
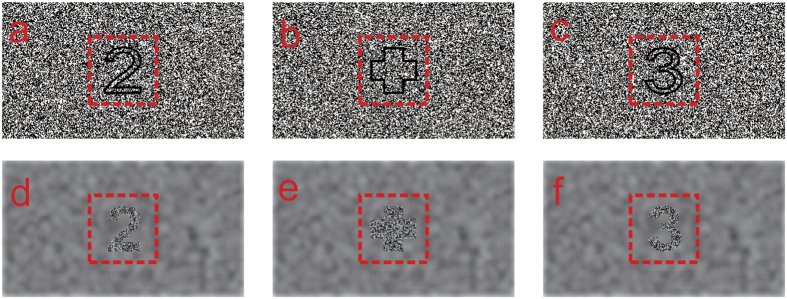
Illustration of the images of the digits and the interval fixation cross in the 2D (**a**–**c**) and 3D (**d**–**f**) conditions. In the 3D condition, the digits (**d**,**f**) and the interval fixation cross (**e**) both possessed a crossed disparity of 0.6 arc deg. In the 2D condition, the profiles of the digits and the interval fixation cross were outlined with fine black line. The square as explained in section 5.2 is indicated by the red dashed box.

In both the 3D and 2D conditions, the subjects were required to wear the polarized glasses and watch the display contents binocularly. The images in the 3D condition were produced based on the random dots stereograms (RDS). The crossed binocular disparity of 0.6 arc deg was adopted according to the review by W. J. Tam^16^ in which this disparity is suggested to be within the most ‘comfort zones’, except for the narrowest one that was thought too rigorous for the dimension of televisions. Therefore the employed disparity was assumed to be well within the ‘comfort zones’ and could provide sufficient sense of depth. Every dot contained 6 × 6 pixels and the duty ratio of the dots was 50%. Because the dynamic RDS would induce the continuous snowstorm (dynamic TV noise) and lead to much sense of flash, an altered approach was used to remove the monocular cues in the image sequence^21^,^39^. A square of 660 × 660 pixels was designed, which was large enough for containing the digits or fixation cross and was at the center of each image. (The square was indicated by the red dashed box in Fig. 3). In order to avoid much sense of flash, the dots array within the square changed between images in the sequence and the dots outside the square kept the same among all images. The 3D image series was viewed as a converting square of dots embedded in a full screen image of static dots, for both monocular and binocular viewing. The digits and the fixation cross were only seen via binocularly fusion of the monocular information from the two eyes, at the center of the square. The images in the 2D condition were similarly constructed but had the zero disparity and the profiles of the digits and the fixation cross were depicted with fine black lines. For both monocular and binocular viewing, the digits and the fixation cross were seen at the center of a converting square of dots embedded in a full screen image of static dots. It should be emphasized that in both the 3D and 2D conditions, the images were binocularly viewed via the polarized glasses and the digits and the fixation cross were seen based on binocular information.

### Procedure

A 23-inch FHD polarized stereoscopic display (LG D2342PY) was used to present the stimuli. The subjects were required to wear the polarized glasses in both the 3D and 2D conditions, sitting 60 cm away from the display. The task of each condition consisted of 600 trials totally, including 120 deviant stimuli (20%) and 480 standard stimuli (80%). The stimulus duration was 150 ms for the digits and was 1.4 s for the interval fixation cross. Before and after each task, the subjective measurement concerning the general state of comfort was conducted with a simple 5-point grading questionnaire, where 1 was for the most comfortable status and 5 was for the least. The oddball task is usually conducted by asking the subjects to respond to the deviant stimuli, either overt (e.g. pressing a key when the deviant stimulus appears) or covert (e.g., counting the number of deviant stimuli)^25^,^30^. S. J. Luck suggests in his book^27^ that responding to both standard and deviant stimuli facilitates eliminating motor confounds in the differential ERP waveforms between the deviant and standard stimuli (i.e., the differential waveforms may be related to a motor confound that subjects make a motor response to the deviant and not the standard stimuli). Therefore, in the present study the subjects were required to respond to both standard and deviant stimuli by pressing the corresponding numeric keys. The EEG signal was recorded from 8 subjects when they performed the tasks. A practice time that was no less than 5-minute was provided before the experiment until the subjects felt confident with the tasks in both conditions. A 20-minute rest was provided between the two conditions. The order of the conditions and the digits indicating the deviant and standard stimuli were counterbalanced among the subjects.

### EEG recording and pre-processing

The EEG was continuously recorded (100-Hz low-pass filtered) and digitized at a rate of 250 Hz by a SynAmps2 64-Channel Quik-Cap system. The horizontal electrooculogram (HEOG) was recorded by two electrodes attached to the left and right outer canthus and the vertical electrooculogram (VEOG) was recorded by two electrodes placed below and above the left eye. The impedance of all electrodes was kept below 5 kΩ (referenced to a default reference electrode placed between CZ and CPZ). The data were re-referenced off-line to the averaged of the left and right mastoid [(LM + RM)/2] potentials. The constant offset was removed from the data, and then the data was filtered by a band-pass filter (0.01 Hz to 15 Hz). After that, the data was segmented into 1200-ms epochs (−200 to 1000 ms relative to the onset of the stimuli). The epochs with a voltage exceeding ±100 μV at any electrode were excluded from analysis.

### ERP analyses

Only the epochs with correct responses were included in the subsequent averaging process. The EEG signal from channel C1 of the participant S3 in the 3D condition was excluded because of bad contact. For both the 2D and 3D conditions, epochs of standard and deviant stimuli were separately averaged. The differential waveforms were obtained by subtracting the average waveforms of the standard stimuli from the average waveforms of the deviant stimuli. First, the topographies of the amplitude of the DP3 signal were obtained for both conditions for the aim of an overall inspection. The most positive peaks in the differential waves were found between 200 ms and 800 ms. The peak times at all electrodes were averaged and the resulting average time was assigned as the peak time of the DP3 signal, which was 441.94 ms (SD = 65.98 ms) for the 2D condition and was 498.84 ms (SD = 110.68 ms) for the 3D condition. The topography of the DP3 signal based on the mean amplitude of a 280-ms time window centered at the DP3 peak time was illustrated for the 2D and 3D conditions (Fig. 2). Subsequently, for statistical analysis, 38 electrodes were selected and divided into 6 groups representing anterior (AL, AR), central (CL, CR) and posterior regions (PL, PR) in the left and right hemispheres. Electrodes at the edge of the net were excluded to reduce contamination from face and neck muscle movement^33^. For the comparisons of ERPs across hemispheres^33^, the ERP data of electrodes in the midline were not presented in the main text and were presented in the Supplementary Materials (see “ERP results of the midline electrodes”. The results were consistent with the results of the six scalp regions reported in the main text). Greenhouse-Geisser corrections were applied to all the repeated measures analysis of variance (ANOVA).

The latency of the DP3 signal in each of the 6 regions in each condition for each participant was defined as the time point of the most positive going peak, which was determined by visually inspection of the corresponding differential waveforms in a time window according to the P3 period of the waveforms of the deviant stimuli.

The amplitude of the DP3 signal in each region in each condition for each participant was defined as following. According to Fig. 2, for each region in each condition, the most positive going peak in the average differential waveform was determined as the peak of the DP3 signal. Given the sampling rate of 250 Hz in the present study, the window width of the DP3 signal was set as 280 ms. The DP3 amplitude was defined as the mean of the signals within the 280-ms window centered at the corresponding peak time of the DP3 signal. Time windows with different widths (240 or 320 ms) were also tested, showing consistent results.

For the trough preceding the DP3 signal, the latency in each region in each condition for each subject was visually determined as the time point of the most negative going peak before the DP3 signal in the corresponding differential waveforms in a time window according to the period of the N2-P3 complex of the waveforms of the deviant stimuli. The amplitude of the trough in each region in each condition for each subject was defined as following. According to Fig. 2, for each region in each condition, the most negative going peak right before the DP3 signal in the differential waveform was determined as the peak of the trough. Given the 250-Hz sampling rate, the window width of the trough was set as 48 ms. The amplitude of the trough was obtained as the mean of the signals within the 48-ms window centered at the corresponding peak time of the trough. Time windows with different widths (40 or 56 ms) were also tested, showing consistent results.

## Additional Information

**How to cite this article:** Ye, P. *et al*. Comparison of DP3 Signals Evoked by Comfortable 3D Images and 2D Images — an Event-Related Potential Study using an Oddball Task. *Sci. Rep.* **7**, 43110; doi: 10.1038/srep43110 (2017).

**Publisher's note:** Springer Nature remains neutral with regard to jurisdictional claims in published maps and institutional affiliations.

## Supplementary Material

Supplementary Information

## Figures and Tables

**Figure 1:**
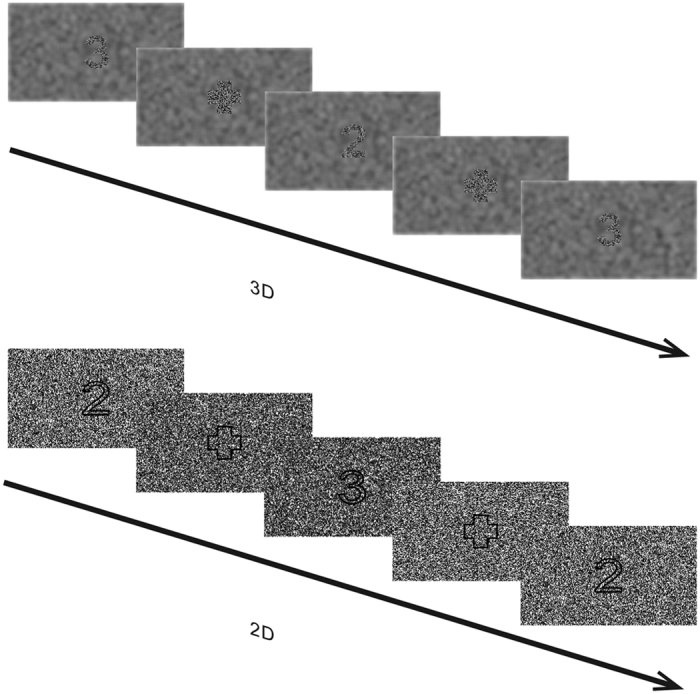
Depiction of the oddball tasks in the 3D and 2D conditions. In each task, the standard (80%) and deviant (20%) stimuli were indicated by different digits (i.e. 2 or 3) and the subjects were required to respond to the stimuli by pressing corresponding keys. Between the digit images is the interval image containing a fixation cross. The upper panel shows the 3D condition where the digits and the interval fixation cross possessed the same crossed binocular disparity of 0.6 arc deg and were viewed as standing in front of the screen. The lower panel shows the 2D condition where the digits and the interval fixation cross were viewed as flat.

**Table 1:** List of the latency and amplitude of the DP3 signal and the comparison between the 2D and 3D condition.

		3D		2D		3D vs. 2D			
		Mean	SD	Mean	SD	Dif Mean	Dif. SD	T value	P value
DP3 Latency (ms)	AL	479.5	83.0	427.0	84.0	52.5	41.7	3.561	**0.009**
	AR	505.5	103.2	432.0	92.8	73.5	61.6	3.373	**0.012**
	CL	519.5	103.3	458.5	68.8	61.0	69.7	2.474	**0.043**
	CR	529.5	103.3	447.5	61.3	82.0	60.7	3.819	**0.007**
	PL	537.5	86.9	468.5	90.4	69.0	105.7	1.847	0.107
	PR	529.0	82.9	448.0	76.7	81.0	112.4	2.039	0.081
DP3 Amplitude (μV)	AL	3.77	2.63	2.18	1.80	1.59	2.35	1.917	0.097
	AR	4.45	2.78	1.81	2.30	2.63	2.67	2.801	**0.026**
	CL	2.68	2.34	2.63	2.05	0.05	1.80	0.08	0.938
	CR	3.70	2.01	2.16	2.05	1.54	1.11	3.932	**0.006**
	PL	0.49	1.05	0.82	1.52	−0.34	1.50	−0.638	0.544
	PR	−0.19	0.84	0.47	1.31	−0.66	1.15	−1.622	0.149

P values smaller than 0.05 are indicated as bold.

**Table 2:** List of the latency and amplitude of the trough and the comparison between 2D and 3D condition.

		3D		2D		3D vs. 2D			
		Mean	SD	Mean	SD	Dif. Mean	Dif. SD	T value	P value
Trough Latency (ms)	AL	367.0	76.4	295.5	44.8	71.5	62.9	3.217	**0.015**
	AR	364.5	70.8	296.0	40.0	68.5	58.6	3.306	**0.013**
	CL	337.5	57.2	307.5	49.4	30.0	34.1	2.485	**0.042**
	CR	342.0	60.8	290.0	35.8	52.0	39.7	3.709	**0.008**
	PL	360.5	57.9	297.0	42.3	63.5	74.9	2.397	**0.048**
	PR	353.0	62.6	297.5	43.5	55.5	75.9	2.069	0.077
Trough Amplitude (μV)	AL	1.08	3.53	−1.41	2.85	2.49	2.03	3.472	**0.01**
	AR	0.80	2.76	−1.46	2.31	2.25	2.00	3.193	**0.015**
	CL	−0.66	2.83	−0.79	3.17	0.12	1.57	0.231	0.824
	CR	−0.75	2.52	−1.02	2.35	0.27	1.48	0.529	0.613
	PL	−3.70	2.46	−2.38	2.89	−1.31	1.60	−2.313	0.054
	PR	−3.38	2.68	−1.95	2.84	−1.42	2.12	−1.906	0.098

P values smaller than 0.05 are indicated as bold.

## References

[b1] Lambooij M., Fortuin M., Heynderickx I. & Ijsselsteijn W. Visual discomfort and visual fatigue of stereoscopic displays: a review. J. Imaging Sci. Technol. 53, 30201–30214 (2009).

[b2] Yano S., Emoto M. & Mitsuhashi T. Two factors in visual fatigue caused by stereoscopic HDTV images. Displays 25, 141–150 (2004).

[b3] Yang S. N. . Stereoscopic viewing and reported perceived immersion and symptoms. Optometry and vision science 89, 1068–1080 (2012). 22733100 10.1097/OPX.0b013e31825da430

[b4] Emoto M., Nojiri Y. & Okano F. Changes in fusional vergence limit and its hysteresis after viewing stereoscopic TV. Displays 25, 67–76 (2004).

[b5] Shibata T., Kim J., Hoffman D. M. & Banks M. S. The zone of comfort: Predicting visual discomfort with stereo displays. Journal of Vision 11, 11–11 (2011). 10.1167/11.8.11 PMC3369815 21778252

[b6] Speranza F., Tam W. J., Renaud R. & Hur N. Effect of disparity and motion on visual comfort of stereoscopic images. Proc. SPIE. 6055, 60550B (2006).

[b7] Li J., Barkowsky M. & Le Callet P. Visual discomfort of stereoscopic 3D videos: Influence of 3D motion. Displays 35, 49–57 (2014).

[b8] Yano S., Ide S., Mitsuhashi T. & Thwaites H. A study of visual fatigue and visual comfort for 3D HDTV/HDTV images. Displays 23, 191–201 (2002).

[b9] Kooi F. L. & Toet A. Visual comfort of binocular and 3D displays. Displays 25, 99–108 (2004).

[b10] Nojiri Y., Yamanoue H., Hanazato A., Emoto M. & Okano F. Visual comfort/discomfort and visual fatigue caused by stereoscopic HDTV viewing. Proc. SPIE 5291, 303–313 (2004).

[b11] Urvoy M., Barkowsky M. & Callet P. How visual fatigue and discomfort impact 3D-TV quality of experience: a comprehensive review of technological, psychophysical, and psychological factors. Ann. Telecommun. 68, 641–655 (2013).

[b12] Hong H. & Kang S. H. Measurement of the lens accommodation in viewing stereoscopic displays. Journal of the Society for Information Display 23, 19–26 (2015).

[b13] Patterson R. Review paper: human factors of stereo displays: an update. Journal of the Society for Information Display 17, 987–996 (2009).

[b14] Inoue T. & Ohzu H. Accommodative responses to stereoscopic three-dimensional display. Appl. Opt. 36, 4509–4515 (1997). 18259243 10.1364/ao.36.004509

[b15] Emoto M., Niida T. & Okano F. Repeated vergence adaptation causes the decline of visual functions in watching stereoscopic television. Journal of Display Technology 1, 328–340 (2005).

[b16] Wa James T., Speranza F., Yano S., Shimono K. & Ono H. Stereoscopic 3D-TV: Visual Comfort. IEEE Transactions on Broadcasting 57, 335–346 (2011).

[b17] Bando T., Iijima A. & Yano S. Visual fatigue caused by stereoscopic images and the search for the requirement to prevent them: A review. Displays 33, 76–83 (2012).

[b18] Chen C. . EEG-based detection and evaluation of fatigue caused by watching 3DTV. Displays 34, 81–88 (2013).

[b19] Mun S., Park M.-C., Park S. & Whang M. SSVEP and ERP measurement of cognitive fatigue caused by stereoscopic 3D. Neuroscience Letters 525, 89–94 (2012). 22884933 10.1016/j.neulet.2012.07.049

[b20] Skrandies W. & Vomberg H. E. Stereoscopic stimuli activate different cortical neurones in man: electrophysiological evidence. International journal of psychophysiology 2, 293–296 (1985). 3997617 10.1016/0167-8760(85)90007-8

[b21] Şahinoğlu B. The effect of disparity change on binocular visual evoked potential parameters elicited by convergent dynamic random-dot stereogram stimuli in humans. European journal of applied physiology 88, 178–184 (2002). 12436288 10.1007/s00421-002-0704-3

[b22] Sahinoglu B. Depth-related visually evoked potentials by dynamic random-dot stereograms in humans: negative correlation between the peaks elicited by convergent and divergent disparities. Eur J Appl Physiol 91, 689–697 (2004). 14704799 10.1007/s00421-003-1028-7

[b23] Omoto S. . Modulation of Human Visual Evoked Potentials in 3-Dimensional Perception After Stimuli Produced With an Integral Imaging Method. Clin. EEG Neurosci. 43, 303–311 (2012). 23185090 10.1177/1550059412445608

[b24] Wijeakumar S., Shahani U., McCulloch D. L. & Simpson W. A. Neural and Vascular Responses to Fused Binocular Stimuli:A VEP and fNIRS Study Responses to Fused Binocular Stimuli. Investigative Ophthalmology & Visual Science 53, 5881–5889 (2012). 22871839 10.1167/iovs.12-10399

[b25] Duncan C. C. . Event-related potentials in clinical research: guidelines for eliciting, recording, and quantifying mismatch negativity, P300, and N400. Clinical Neurophysiology 120, 1883–1908 (2009). 19796989 10.1016/j.clinph.2009.07.045

[b26] Polich J. Updating P300: an integrative theory of P3a and P3b. Clinical neurophysiology 118, 2128–2148 (2007). 17573239 10.1016/j.clinph.2007.04.019 PMC2715154

[b27] Luck S. J. An Introduction to the Event-Related Potential Technique (MIT press, 2014).

[b28] Kutas M., McCarthy G. & Donchin E. Augmenting mental chronometry: the P300 as a measure of stimulus evaluation time. Science 197, 792–795 (1977). 887923 10.1126/science.887923

[b29] Kok A. On the utility of P3 amplitude as a measure of processing capacity. Psychophysiology 38, 557–577 (2001). 11352145 10.1017/s0048577201990559

[b30] Johnson R. The amplitude of the P300 component of the event-related potential: Review and synthesis. Advances in psychophysiology 3, 69–137 (1988).

[b31] Patel S. H. & Azzam P. N. Characterization of N200 and P300: selected studies of the event-related potential. International Journal of Medical Sciences 2, 147 (2005). 16239953 10.7150/ijms.2.147 PMC1252727

[b32] Rouder J. N., Speckman P. L., Sun D., Morey R. D. & Iverson G. Bayesian t tests for accepting and rejecting the null hypothesis. Psychonomic Bulletin & Review 16, 225–237 (2009). 19293088 10.3758/PBR.16.2.225

[b33] Hove M. J., Marie C., Bruce I. C. & Trainor L. J. Superior time perception for lower musical pitch explains why bass-ranged instruments lay down musical rhythms. Proceedings of the National Academy of Sciences 111, 10383–10388 (2014). 10.1073/pnas.1402039111 PMC4104866 24982142

[b34] Gray H. M., Ambady N., Lowenthal W. T. & Deldin P. P300 as an index of attention to self-relevant stimuli. Journal of Experimental Social Psychology 40, 216–224 (2004).

[b35] Lew G. S. & Polich J. P300, habituation, and response mode. Physiol Behav 53, 111–117 (1993). 8434050 10.1016/0031-9384(93)90018-b

[b36] Romero R. & Polich J. P3(00) habituation from auditory and visual stimuli. Physiology & Behavior 59, 517–522 (1996). 8700955 10.1016/0031-9384(95)02099-3

[b37] Ravden D. & Polich J. Habituation of P300 from visual stimuli. International Journal of Psychophysiology 30, 359–365 (1998). 9834892 10.1016/s0167-8760(98)00039-7

[b38] John E. H. & Frank L. S. Methods of Meta-Analysis: Correcting Error and Bias in Research Findings (SAGE Publications, Inc. 2nd edition 2004).

[b39] Lehmann D. & Julesz B. Lateralized cortical potentials evoked in humans by dynamic random-dot stereograms. Vision Research 18, 1265–1271 (1978). 726269 10.1016/0042-6989(78)90216-x

