# Supplementary material for: Comparison of DP3 Signals Evoked by Comfortable 3D Images and 2D Images — an Event-Related Potential Study using an Oddball Task
Source: Sci Rep. 2017 Feb 22;7:43110. doi: 10.1038/srep43110 (PMC5320480; doi:10.1038/srep43110)
Supplement: Supplementary Information [file srep43110-s1.pdf]

Comparison of DP3 Signals Evoked by Comfortable 3D Images and 2D Images — an Event-Related Potential Study using an Oddball Task

Peng Ye[1,2], Xiang Wu[3,*], Dingguo Gao[3], Haowen Liang[2], Jiahui Wang[2], Shaozhi Deng[1,2], Ningsheng Xu[1,2], Juncong She[1,2], Jun Chen[1,2*]

[1] State Key Laboratory of Optoelectronic Materials and Technologies, Guangdong Province Key Laboratory of Display Material and Technology, School of Electronics and Information Technology, Sun Yat-sen University, Guangzhou, China

[2] State Key Laboratory of Optoelectronic Materials and Technologies, Sun Yat-Sen University, Guangzhou, China

[3] Department of Psychology, Sun Yat-Sen University, Guangzhou, China

[*] Email: stscjun@mail.sysu.edu.cn; rwfwuwx@gmail.com

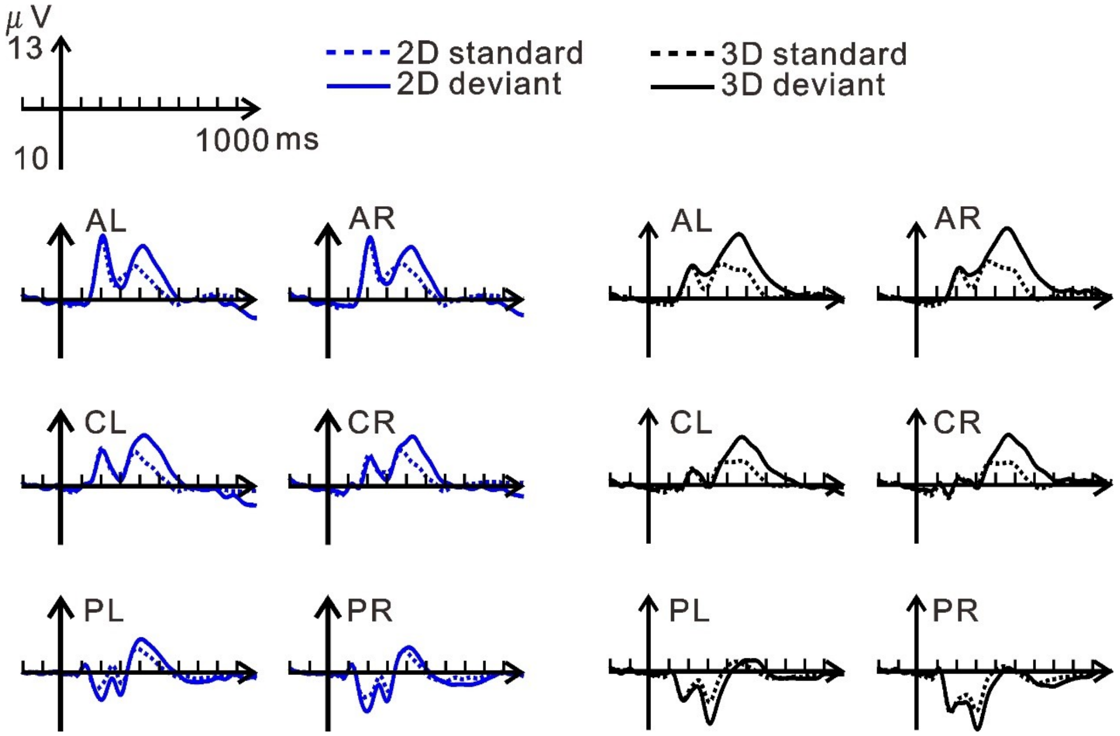

Supplementary Figure S1. Illustration for the average raw waveforms of the deviant and standard stimuli in each condition. Conventions are as in Figure 2.

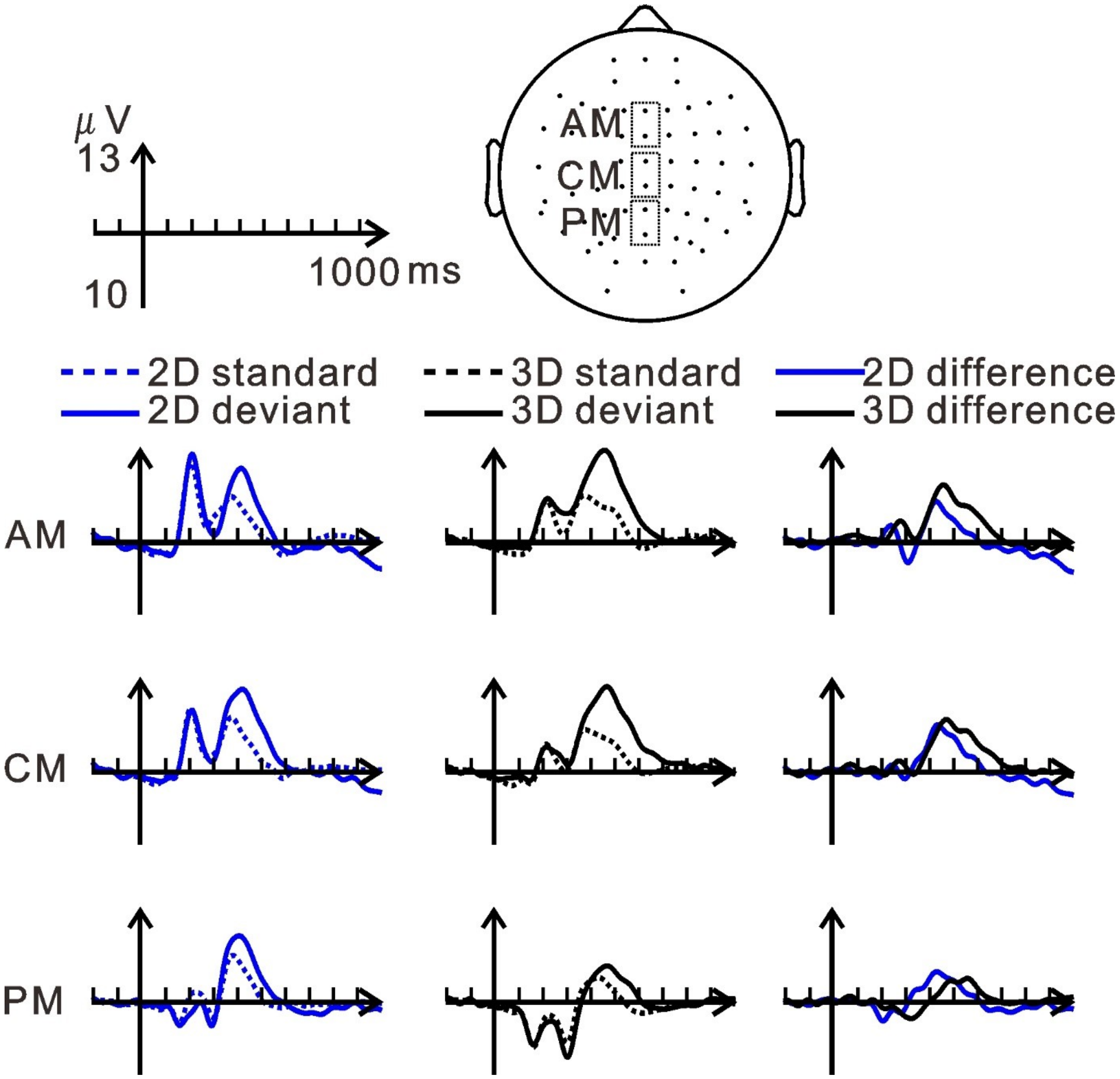

Supplementary Figure S2. Illustration for the average raw waveforms of the deviant and standard stimuli in each condition. Shown are the results of the mid-line electrodes, which were clustered into 3 scalp regions (anterior (AM), central (CM), posterior (PM)). Conventions are as in Figure S1.

Supplementary Table S1. DP3 latency for each participant. The DP3 signal had longer latency in the 3D than in the 2D condition for all participants at all the 6 regions, except for participant S8 at Region CL, PL and PR and participant S5 at Region PR (indicated as blue).

	DP3 latency (ms)											
	AL		AR		CL		CR		PL		PR	
Subjects	2D	3D	2D	3D	2D	3D	2D	3D	2D	3D	2D	3D
S1	516	620	512	676	524	680	520	680	608	680	528	680
S2	512	584	560	584	520	580	524	584	528	584	380	588
S3	436	440	436	440	440	456	436	460	424	460	428	468
S4	452	460	456	600	456	552	464	600	468	504	472	516
S5	416	456	420	468	424	440	432	456	408	424	432	412
S6	252	368	248	376	320	364	328	372	316	624	324	560
S7	380	428	380	420	460	608	444	608	456	540	456	536
S8	452	480	444	480	524	476	432	476	540	484	564	472

Supplementary Table S2. List of the latency and amplitude of the DP3 signal and the trough, as well as the corresponding comparison between the 2D and 3D condition. Shown are the results of the mid-line electrodes, which were clustered into 3 scalp regions (anterior (AM), central (CM), posterior (PM)). P values smaller than 0.05 are indicated as yellow.

		3D		2D		3D vs. 2D			
		Mean	SD	Mean	SD	Dif-Mean	Dif-SD	T-value	P-value
DP3 latency (ms)	AM	478.5	79.6	424.5	81.9	54	41.3	3.69	0.008
	CM	487.5	95.7	446.5	59.8	41	53.1	2.19	0.065
	PM	529	83.5	446	70.4	83	101.1	2.32	0.053
DP3 amplitude (µV)	AM	4.66	3.5	2.02	2.42	2.64	3.43	2.18	0.066
	CM	4.53	3.56	3.4	2.75	1.13	2.05	1.56	0.16
	PM	1.82	1.55	2.23	2.74	-0.41	1.63	-0.7	0.51
Trough latency (ms)	AM	364	70.8	316.5	70.7	47.5	37.1	3.62	0.009
	CM	359.5	70.4	294	82.8	65.5	56.9	3.26	0.014
	PM	351.5	61.9	282.5	51.4	69	70.1	2.78	0.027
Trough amplitude (µV)	AM	0.80	3.69	-2.24	3.18	3.04	2.9	2.96	0.02
	CM	0.03	3.37	-0.53	2.9	0.56	1.8	0.88	0.41
	PM	-2.14	2.65	-2.2	1.93	0.06	2.89	0.06	0.95

ERP Results of the Midline Electrodes

For the DP3 signal

The results are shown in Supplementary Figure S2. For the latency, the repeated measures analysis of variance (ANOVA) showed a significant main effect of dimension [$F(1,7)=8.98$, $P<0.05$, $\eta^2=0.562$]. For the amplitude, the ANOVA result showed a significant main effect of region [$F(2,14)=4.12$, $P<0.05$, $\eta^2=0.371$] and a marginal effect of the interaction of dimension×region [$F(2,14)=3.7$, $P=0.089$, $\eta^2=0.346$]. No other significance was found. The post-hoc comparisons for the DP3 amplitude between the 3D and 2D conditions are listed in Supplementary Table S2. The results were consistent with the results of the six scalp regions reported in the main text, showing that the DP3 signal in the 3D condition had delayed latency and enhanced amplitude over anterior and central scalp regions compared to the 2D condition.

For the Trough before the DP3 Signal

For the latency, the ANOVA result showed a significant main effect of dimension [$F(1,7)=20.97$, $P<0.005$, $\eta^2=0.75$]. For the amplitude, the significant main effects of dimension [$F(1,7)=9.72$, $P<0.05$, $\eta^2=0.581$] and region [$F(2,14)=4.62$, $P<0.05$, $\eta^2=0.397$] were found. In addition, there was a weak interaction of dimension×region [$F(2,14)=2.48$, $P=0.15$, $\eta^2=0.261$]. No other significance was found. The post-hoc comparisons for the amplitude of the trough are listed in Supplementary Table S2. The results were consistent with the results of the six scalp regions reported in the main text, showing that the trough in the 3D condition was of longer peak latency and had larger peak amplitude over anterior scalp regions compared to the 2D conditions.
